# Initial Effects of Electroacupuncture for Chronic Severe Functional Constipation and the Potential Underlying Factors: Secondary Analysis of a Randomized Controlled Trial

**DOI:** 10.1155/2019/7457219

**Published:** 2019-05-06

**Authors:** Yuxiao Zeng, Yan Liu, Sixing Liu, Zhishun Liu

**Affiliations:** ^1^Department of Acupuncture, Guang'anmen Hospital, China Academy of Chinese Medical Sciences, Beijing 100053, China; ^2^China Academy of Chinese Medical Sciences, Beijing 100700, China; ^3^Institute of Basic Research in Clinical Medicine, China Academy of Chinese Medical Sciences, Beijing 100700, China; ^4^College of Acupuncture and Orthopedics, Guiyang University of Chinese Medicine, Guiyang 550000, China

## Abstract

**Background:**

Electroacupuncture (EA) has been found to be effective for treating chronic severe functional constipation (CSFC). However, the initial effects of treatment usually affect the acceptability and compliance of patients with chronic disease in particular. Which class of CSFC patients will have a better initial response to EA remains uncertain and requires investigation.

**Methods:**

This was a secondary analysis of an original multicenter randomized controlled trial in which patients with CSFC were randomly assigned to receive 28 sessions of EA or sham electroacupuncture (SA) over 8 weeks with 12 weeks of follow-up. The primary outcome, namely, response with complete spontaneous bowel movements (CSBMs), required participants to have ≥ 3 CSBMs and an increase of ≥ 1 CSBM from the baseline over the first week of treatment. Logistic regression analysis with bootstrapping techniques was performed to determine independent factors related to the response.

**Results:**

A total of 1051 eligible patients were included in this study of whom 161 patients were classified as responders at week 1. The CSBM response rate was higher in the EA group (17.5%) than in the SA group (13.2%). And the proportion of these 1-week early responders remained to have higher clinical response at the end of 8-week treatment and 12 weeks after treatment. Age and higher baseline CSBMs were related to CSBM response within the first week: with every 1-year increase in age, the likelihood of clinical response was reduced by 1.7% (odds ratio [OR] 0.983, 95% confidence interval [CI] 0.972 to 0.993; P=0.001). The odds of a CSBM response in patients with 1< CSBMs ≤ 2 at baseline were 4.64 times higher than that in patients with CSBMs ≤ 1 (OR 4.64, 95%CI 4.01 to 5.27).

**Conclusions:**

EA produced its initial effects within the first week of treatment. And the effects could last until week 8 and week 20. A younger age and higher number of CSBMs at baseline may increase likelihood of a response.

## 1. Introduction

Chronic constipation is a common gastrointestinal disorder. According to recent epidemiological data, the prevalence of chronic constipation ranges from 2% to 27% in North America [1]. Chronic constipation brings about a psychological burden, affects relationships, lowers physical productivity, and decreases the individual's quality of life [2]. It is estimated that nearly 50% of patients suffering from constipation were not completely satisfied with the pharmacological treatment they had received, including fiber and laxatives, owing to safety concerns or lack of efficacy [3]. The initial effects of a treatment could affect its acceptability and compliance to the treatment, especially for patients afflicted with chronic diseases, which has been widely considered as exerting a critical influence on the effectiveness of medical interventions [4, 5].

A previous randomized, sham-controlled, multicenter trial on using acupuncture for CSFC showed that EA increased the mean number of CSBMs each week over 8 weeks of treatment [6]. Because the original study mainly examined whether acupuncture was effective for chronic constipation, the initial effects of acupuncture and the influential factors associated with it remain largely unknown.

To address the limitations of previous research, a secondary analysis was performed using strict outcome measures to explore the initial effect of EA and to identify the associated factors affecting the response of patients with CSFC to EA.

## 2. Methods

### 2.1. Overview of the Original Trial

The specific details of the original trial were included in the protocol, which we have published [7]. The original study was a randomized, sham-controlled, parallel, multicenter trial conducted at 15 sites in China. A total of 1075 patients were recruited and assigned using stratified block randomization to the EA group (n = 536) or the sham electroacupuncture (SA) group (n = 539). Fifty-four patients dropped out during the course of the study. The study duration per patient was 22 weeks, including 2 weeks before randomization (baseline assessment), 8 weeks of treatment, and 12 weeks of follow-up without treatment. The primary outcome was the change from the baseline in mean weekly CSBMs from weeks 1 to 8. The secondary outcomes included changes from the baseline in mean spontaneous bowel movements per week, mean score on the Bristol stool form scale, mean score from patient's assessment of constipation quality of life (PAC-QOL) [8], the proportion of participants with 3 or more mean CSBMs per week, and the proportion of participants using emergency medicine and other defection methods for constipation. Adverse events throughout the whole trial were also assessed.

The study protocol had been approved by all 15 members of the local Ethics Committee prior to the investigation, and the trial was registered at ClinicalTrials.gov (NCT01726504).

### 2.2. Secondary Analysis Design

In this study, we explored the initial effect of EA after treatment and identified the associated factors. The European Medicines Agency's guideline indicates that the use of a primary endpoint based on CSBMs is acceptable because it incorporates spontaneity and completeness of the bowel movement and recommends a responder analysis [9]. A weekly CSBM responder was defined as a patient who had at least 3 CSBMs/week and experienced an increase of at least 1 CSBM/week compared to the baseline in the same time [9]. Therefore, the primary outcome of this secondary analysis was the CSBM responder rate within the first week. Responders and nonresponders were classified according to whether they had ≥ 3 CSBMs and a ≥ 1 increase from baseline for the first week after treatment. And these 1-week early responders were continued to be tracked at the end of 8-week treatment and 12 weeks after treatment. We also analyzed the proportion of patients with CSBMs within the first 24 hours of treatment, and the number of days taken by patients to have the first CSBM.

Factors related to the initial effect of EA were also assessed. We analyzed the baseline characteristics of the patients, including treatment assignment, age, race, body mass index (BMI), and some indicators associated with CSFC, such as duration of constipation, CSBMs per week, PAC-QOL score, and comorbidities. The mean CSBMs per week at baseline indicated the patients' bowel function. The mean PAC-QOL score indicated the effects of constipation on physical discomfort, psychosocial discomfort, worriedness, concerns, and satisfaction in their daily lives.

### 2.3. Statistical Analysis

The primary outcome was analyzed using a generalized linear model with a binomial distribution, adjusted for sites. The same approach was used for the proportion of patients with CSBMs within the first 24 hours of treatment.

Descriptive statistics were used to compare the demographics and baseline characteristics between the responder and nonresponder groups. All variables with P values of <0.25 from the univariate analysis [10] were considered as potential candidates for the multivariate logistic regression model. The model also included the interactions between the treatment assignment and the candidate variables. Then backward elimination with 1,000 bootstrap samples as a variable selection strategy was used to retain these variables in the final model [11]. The variance inflation factor (VIF) was used to detect multicollinearity among the independent variables before the regression analyses. Multicollinearity was considered if the VIF for one of the variables exceeded 5.

Analyses were based on the intention-to-treat principle, with all randomly assigned participants included. A two-sided P value < 0.05 was considered statistically significant. All statistical tests were performed in SAS 9.4 (SAS Institute, Cary NC) and R version 3.4.1 (The R Foundation for Statistical Computing, Vienna, Austria).

## 3. Results

### 3.1. CSBM Response Rate and Mean Time to First CSBM

A total of 1051 participants were included in this study, of whom 161 (15.3%) were classified as responders at week 1. The baseline characteristics of the whole population have been described in our previous original research paper [6]. Regarding the primary outcome, Table 1 displays the CSBM response rates were 17.5% in the EA group and 13.2% in the SA group during the first week of treatment. The proportion of patients with CSBMs within the first 24 hours of treatment was significantly higher in the EA group than in the SA group (14.6% versus 10.1%). And the mean number of days that elapsed before the first CSBM was 8.4±10.6 in the EA group and 8.5±11.6 in the SA group. And Table 2 shows the higher response rates of these 1-week early responders could last until week 8 (82.2% versus 52.3%) and week 20 (67.8% versus 38.5%).

### 3.2. Logistic Regression Analysis of Related Factors in Responders

The baseline characteristics of the responders are shown in Table 3, including their demographic and clinical characteristics. Logistic regression analysis with backward elimination identified the following: (i) 4 of the 9 factors (5 candidate variables and 4 interactions between the candidate variables and group) as significantly related to CSBM response: namely, group, age, and baseline CSBMs, and (ii) the interaction between group and baseline CSBMs (Tables 4 and 5). The odds of CSBM response in the EA group were 2-fold higher than those in the SA group (OR 0.500, 95%CI 0.113 to 0.886; P< 0.001). Increased age was associated with nonresponse, with every 1-year increase in age, and the likelihood of clinical response was decreased by 1.7% (OR 0.983, 95%CI 0.972 to 0.993; P=0.001); the odds of CSBM response in patients with CSBMs > 1 to ≤ 2 at baseline were 4.64 times higher than those in patients with CSBMs ≤ 1. The results showed that younger patients and patients with more baseline CSBMs had higher response rates. The effects of interactions also showed that patients with more CSBMs at baseline had a better response to EA (Table 4).

## 4. Discussion

The results of the present study showed that EA brought about, after the first week of treatment, a higher proportion of CSBM responders among patients with CSFC. And the effects could last until week 8 and week 20. Moreover, EA, a younger age and a higher number of CSBMs at baseline were associated with a greater likelihood of CSBM response within the first week of treatment. Factors such as race, BMI, constipation duration, PAC-QOL score, and comorbidity were found to have no influence on the response to EA.

In accordance with the recommendation of the European Medicines Agency's guideline, we chose CSBM response rate as the primary outcome. After the first week of treatment, the CSBM response rates were 17.5% in the EA group and 13.2% in the SA group. A previous randomized control trial showed that after 1 week of treatment, the response rates in the 3 mg plecanatide group, 6 mg plecanatide group, and placebo group were 35.8%, 29.3%, and 16.6%, respectively [12]. In another trial, after 1 week of treatment, the proportions of patients with functional constipation whose defecation frequency had increased to 4 times per week were 64.58%, 66.67%, and 70.83%, respectively, in groups undergoing deep needling, shallow needling, or receiving medication. The effects on increasing the number of defecation per week were similar in the needling group and the medication group [13], which was similar to the findings from our analysis. The participants included in these two studies were not limited to chronic constipation sufferers with only two or fewer CSBMs per week, as the subjects in our trial were. This difference in the study populations may be responsible for the discrepancy in the results.

Based on previous studies, the occurrence of constipation is related to gender, age, and race. The incidence of constipation in women, non-whites, children, and the elderly is relatively high [14–16]. In addition, physical inactivity, low income, limited education, a history of sexual abuse, and depression are all risk factors for constipation [17]. Also, factors that affect the therapeutic effects of acupuncture are complicated, including both specific and nonspecific factors such as needle insertion, acupoint specificity, acupuncture manipulation, stimulation parameters, needle duration, and treatment interval [18, 19]. This secondary analysis from Table 4 showed that the factors influencing effectiveness of EA within the first week of treatment for CSFC were group, age, and CSBMs at baseline, while responders and nonresponders showed no differences in race, BMI, constipation duration, PAC-QOL score, and comorbidity. Some studies have demonstrated that colonic motility changes in both human and animals with increasing age [20, 21]. Constipation in elderly patients is also related to other internal and external factors, such as pelvic floor aging, decreased social activity, psychological disorders, comorbidity, and the effects of multiple drug usage [22]. Therefore, elderly patients have a poorer response to EA. As this trial was conducted in China, race comparison was performed mainly between Han and other ethnic minorities who are all of Chinese ethnicity. To date, there is not much research showing differences in the response between Han Chinese and minorities. BMI also may be associated with the occurrence of constipation [23, 24], but no study has shown a relationship between the curative effect of EA and BMI. There were no differences between responders and nonresponders regarding factors related to the severity of chronic constipation, such as duration of constipation, PAC-QOL score, and comorbidities. Nevertheless, the results in Table 3 show that there were more responders among patients with a duration of constipation of 10 years or less than among patients with a duration of constipation exceeding 10 years. There also were more responders with a PAC-QOL score ≤ 2 than with a PAC-QOL score > 2. Additionally, although previous analysis indicated that it was more likely for patients without a comorbidity than patients with a comorbidity to be responders by week 20 [25], comorbidity appeared to have no effect on the efficacy of the first week of EA treatment.

To the best of our knowledge, this is the first study to assess the initial effectiveness and risk factors of EA for patients with chronic constipation. However, there are still limitations in this secondary analysis. We analyzed only some of the demographic factors. Some potential factors including mental and psychological status, eating habits, educational level attained, geographical environment, and occupation may also affect the curative effect of EA within the first week of treatment. In addition, other possible factors like acupoints, acupuncture occasion, acupuncture manipulation, and stimulation volume deserve further investigation as well.

## 5. Conclusions

EA produces a certain initial effect on patients with CSFC in the first week of treatment. And the effects could last until week 8 and week 20. A younger age and higher number of CSBMs at baseline may be associated with a better response to EA treatment.

## Figures and Tables

**Table 1:** Efficacy of EA in patients with CSFC^a^.

Characteristics	EA group	SA group	Total	P-value
	(n=527)	(n=524)	(N=1051)	
Patients with CSBM response within the first week of treatment^b^	92 (17.5)	69 (13.2)	161 (15.3)	0.054

Patients with CSBM within the first 24 h of treatment	77 (14.6)	53 (10.1)	130 (12.4)	0.026

Time to first CSBM, days^c^				
Mean (SD)	8.4 (10.6)	8.5 (11.6)	8.4 (11.2)	
Median (IQR)	4 (1-12)	3 (1-11)	4 (1-12)	

EA: electroacupuncture; SA: sham electroacupuncture; CSBM: complete spontaneous bowel movement; IQR: interquartile range.

^a^Data are expressed as number of participants (%) unless otherwise indicated; 24 participants missed the 1-wk defecation diaries but completed all other defecation diaries (9 in the EA group and 15 in the SA group).

^b^A CSBM weekly response was defined as a patient who had ≥3 CSBMs for a given week and an increase from the baseline of ≥1 CSBM for the same week.

^C^Data analysis was not performed due to descriptive purposes only.

**Table 2:** The carry-over effect of the 1-week early responders^a^.

Characteristics	EA group	SA group	Total	P-value
Patients with CSBM response within the first week of treatment				
Week 8	74/90(82.2)	34/65(52.3)	108/155(69.7)	<0.001
Week 20	61/90(67.8)	25/65(38.5)	86/155(55.5)	<0.001

Patients with CSBM non-response within the first week of treatment				
Week 8	199/428(46.5)	73/442(16.5)	272/870(31.3)	<0.001
Week 20	160/425(37.7)	60/441(13.6)	220/866(25.4)	<0.001

^a^The number of patients with CSBM response and nonresponse within the first week of treatment are derived from Table 1.

**Table 3:** Demographic and clinical characteristics of CSBM responders within the first week of EA treatment^a^.

Characteristics	Non-responders	Responders	Total	P-value^b^
	(n=890)	(n=161)	(N=1051)	
Age, mean (SD), y	47.9 (15.91)	42.5 (16.63)	47.1 (16.13)	<0.001
Race				0.295
Han	863 (97.0)	159 (98.8)	1022 (97.2)	
Non-Han	27 (3.0)	2 (1.2)	29 (2.8)	
BMI (kg/m2)				0.419
≤18.5	53 (6.0)	14 (8.7)	67 (6.4)	
>18.5 to ≤23.9	573 (64.4)	104 (64.6)	677 (64.4)	
>23.9 to ≤27.9	229 (25.7)	35 (21.7)	264 (25.1)	
>27.9	35 (3.9)	8 (5.0)	43 (4.1)	
Constipation duration, y				0.224
≤10	566 (63.6)	118 (73.3)	684 (65.1)	
>10 to ≤20	196 (22.0)	27 (16.8)	223 (21.2)	
>20 to ≤30	84 (9.4)	12 (7.5)	96 (9.1)	
>30 to ≤40	31 (3.5)	3 (1.9)	34 (3.2)	
>40 to ≤50	9 (1.0)	0 (0.0)	9 (0.9)	
>50	4 (0.4)	1 (0.6)	5 (0.5)	
CSBMs				<0.001
≤1	835 (93.8)	106 (65.8)	941 (89.5)	
>1 to ≤2	55 (6.2)	55 (34.2)	110 (10.5)	
PAC-QOL score				<0.001
≤2	127 (14.3)	46 (28.6)	173 (16.5)	
>2 to ≤3	468 (52.6)	66 (41.0)	534 (50.8)	
>3 to ≤4	259 (29.1)	41 (25.5)	300 (28.5)	
>4 to ≤5	36 (4.0)	8 (5.0)	44 (4.2)	
Comorbidity				0.543
Yes	196 (22.0)	32 (19.9)	228 (21.7)	
No	694 (78.0)	129 (80.1)	823 (78.3)	

BMI: body mass index; CSBM: complete spontaneous bowel movement; PAC-QOL: patient assessment of constipation quality of life.

^a^Data are expressed as no. of participants (%) unless otherwise indicated; 24 participants missed the 1 wk defecation diaries but completed all other defecation diaries (9 in the EA group and 15 in the SA group).

^b^A threshold of P<0.25 was used to select variables [10].

**Table 4:** Backward logistic regression with bootstrap method.

Variables	B	SE	P-value	Odds Ratio (95%CI)^a^
Intercept	-0.980	0.265		
Age	-0.017	0.005	0.001	0.983(0.972 to 0.993)
CSBMs	1.535	0.323	<0.001	4.641(4.014 to 5.268)
Treatment assignment	-0.694	0.197	<0.001	0.500(0.113 to 0.886)
CSBMs*∗*Treatment assignment	0.961	0.438	0.028	2.615(1.756 to 3.474)

^a^Regression coefficient and corresponding odds ratio after bootstrapping (i.e., adjusted for overfitting).

**Table 5:** Details in the interaction between group and baseline CSBMs.

CSBMs	EA group (n=527)		SA group (n=524)	
	Responders	Non-responders	Responders	Non-responders
≤1	70(76.1)	410(94.5)	36(52.2)	425(93.4)
>1 to ≤2	22(23.9)	25(5.75)	33(47.8)	30(6.59)

## Data Availability

The data used to support the findings of this study are included within the article.
